# Intestinal epithelial SETD2 maintains gut microbial homeostasis to attenuate colitis

**DOI:** 10.1002/ctm2.70754

**Published:** 2026-08-04

**Authors:** Jing Feng, Ziyi Wang, Yue Xu, Jinjin Peng, Che Xu, Qingxin Xie, Yuyi Li, Wangshuang Chen, Jiaqing Chen, Xingyu Wang, Wei‐Qiang Gao, Li Li, Xiangjun Meng

**Affiliations:** ^1^ Department of Gastroenterology, Shanghai Ninth People's Hospital, School of Medicine Shanghai Jiao Tong University Shanghai China; ^2^ Shanghai Key Laboratory of Gut Microecology and Associated Major Diseases Research Shanghai China; ^3^ Digestive Disease Research and Clinical Translation Center Shanghai Jiao Tong University Shanghai China; ^4^ State Key Laboratory of Systems Medicine for Cancer, Renji‐Med X Clinical Stem Cell Research Center, Ren Ji Hospital, School of Medicine and School of Biomedical Engineering Shanghai Jiao Tong University Shanghai China; ^5^ School of Biomedical Engineering and Med‐X Research Institute Shanghai Jiao Tong University Shanghai China

**Keywords:** AMPs, epigenetics, gut microbes, IBD, SETD2

## Abstract

**Background:**

Disruption of host–microbiota homeostasis is a fundamental hallmark of inflammatory bowel disease (IBD) pathogenesis. Host epigenetic modifications and corresponding alterations in gene expression levels can impact the composition of gut microbes. SET domain containing 2 (SETD2) is a critical epigenetic regulator with established tumor‐suppressive roles, but its function in intestinal microbial ecology and colitis progression remains unexplored. We aimed to investigate the specific role of SETD2 in maintaining gut microbial homeostasis and modulating colitis progression.

**Methods:**

RNA sequencing (RNA‐seq), assay for transposase‐accessible chromatin with high‐throughput sequencing (ATAC‐seq) and cleavage under targets and tagmentation sequencing (CUT&Tag‐seq) were conducted on colonic epithelial cells from intestinal epithelial cell‐specific SETD2 knockout (Setd2^vil‐ko^) mice to identify key mediators contributing to colitis development. Faecal samples underwent 16S rRNA sequencing and non‐targeted metabolomics analysis to characterise microbial dysbiosis and metabolic perturbations. Molecular experiments and faecal microbiome transplantation experiment were conducted to explore and validate the role of SETD2 in colitis development.

**Results:**

SETD2 deficiency induced overproduction of Reg3 lectins and disrupted gut microbiota composition. Beneficial commensal bacteria were depleted and dysregulated metabolites were accumulated in Setd2^vil‐ko^ mice. Supplementation with healthy‐like gut microbiota significantly ameliorated the exacerbated colitis induced by SETD2 deficiency.

**Conclusions:**

Our findings uncover a previously unrecognised role for SETD2 in maintaining microbial homeostasis, offering new mechanistic insights into how epigenetic regulation preserves intestinal homeostasis and suggesting novel therapeutic avenues for IBD.

## Highlights

- SETD2 deficiency in intestinal epithelial cells exacerbates colitis and drives the aberrant overespression of the antimicrobial peptides REG3.
- SETD2 deficiency promotes REG3 expression by increasing chromatin accessibility and enhancing H3K4me3 enrichment ad *Reg3* loci.
- SETD2 deficiency induces gut microbiota dysbiosis and aberrant metabolite accumulation.

## Graphical abstract

• SETD2 deficiency in intestinal epithelial cells (IECs) drives REG3 lectins overproduction and triggers profound gut microbiota dysbiosis.

• SETD2 deficiency increased chromatin accessibility and induced H3K4me3 enrichment at REG3 genes.

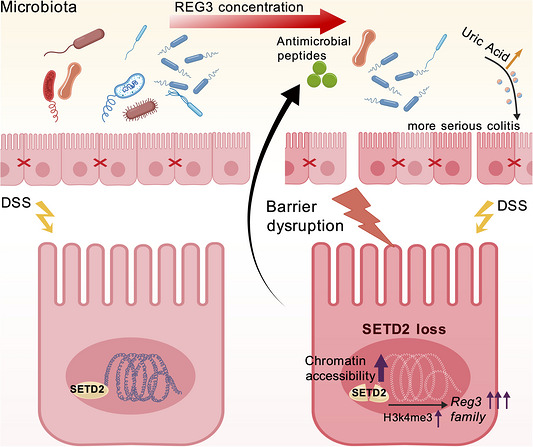

## 1 INTRODUCTION

Inflammatory bowel disease (IBD), encompassing Crohn's disease (CD) and ulcerative colitis (UC), is a chronic relapsing disorder of the gastrointestinal tract. Despite the aetiology of IBD remains unclear, mounting evidence indicates that IBD results from the complex interplay of genetic susceptibility, gut microbial dysbiosis, environmental triggers, intestinal barrier dysfunction and dysregulated immune responses.^1^, ^2^ Disruption of host–microbiota homeostasis is a central hallmark of IBD pathogenesis. This imbalance involves the depletion of beneficial commensals and the expansion of pro‐inflammatory pathobionts, which subsequently compromises epithelial integrity and perpetuates mucosal inflammation.^3^, ^4^

Epigenetic modifications are increasingly recognised as critical mediators linking integrate genetic risk with environmental factors to modulate IBD susceptibility and progression.^5^, ^6^ Genome‐wide association studies have identified over 300 susceptibility loci associated with IBD, many of which reside in non‐coding regulatory regions subject to epigenetic control.^7^ Aberrant epigenetic alterations, including dysregulated DNA methylation, histone modifications and non‐coding RNA expression, have been demonstrated to drive chronic inflammation by disrupting immune cell differentiation and compromising epithelial repair mechanisms.

SET domain containing 2 (SETD2) is a pivotal epigenetic regulator, functioning as the primary histone methyltransferase responsible for catalysing the tri‐methylation of lysine 36 on histone H3 (H3K36me3). Originally characterised as a tumour suppressor in renal and haematologic malignancies,^8^, ^9^, ^10^ SETD2 has recently emerged as a pivotal player in inflammation regulation. Our previous work demonstrated that intestinal epithelium‐specific deletion of SETD2 exacerbates colitis in mice, associated with impaired antioxidant responses and compromised mucosal healing.^11^ However, the complete network of regulatory pathways utilised by SETD2 to maintain intestinal homeostasis and limit inflammation remains to be fully elucidated.

Recent studies have redefined the paradigm of host‒microbe interactions from a unidirectional model to a dynamic interplay, wherein the host's epigenetic status actively modulates the gut ecosystem.^12^, ^13^ The host can intrinsically regulate microbial homeostasis through its own epigenetic machinery, which includes transcriptional modulation of immune‐related genes to govern inflammatory responses and immune cell differentiation,^14^ sustaining the structural integrity and selective permeability of the intestinal epithelial barrier,^15^ and epigenetic modulation of microbial metabolic activity via noncoding RNA pathways.^16^, ^17^ In addition, compelling experimental evidence have established that host epigenetic modifications and corresponding alterations in gene expression levels significantly impact the composition of the microbial community.^18^, ^19^, ^20^, ^21^ However, whether epigenetic regulator SETD2 can impact gut microbiota remains unexplored. Therefore, it is essential to investigate the specific role of SETD2 in maintaining gut microbial homeostasis and modulating the progression of colitis.

In present study, we performed a detailed analysis of the intestinal transcription and faecal microbiome on the intestinal epithelial cell (IEC)‐specific SETD2 knockout mice. Our data show that SETD2 deficiency can promote the overexpression of key antimicrobial peptides (AMPs) and lead to dysbiosis of the gut microbiota and metabolites. Our findings uncover a previously unrecognised role for SETD2 in maintaining microbial homeostasis, offering new mechanistic insights into how epigenetic regulation preserves intestinal homeostasis and suggesting novel therapeutic avenues for IBD.

## 2 METHODS

### 2.1 Mouse models

Setd2^f/f^ and Setd2^vil‐ko^ mice were generated as previously described.^11^ All genetically modified mice were on a C57BL/6J background. Mice used in the following experiments were sex‐ and age‐matched. Experimental procedures were approved by the Animal Care and Use Committee of Shanghai Ninth People's Hospital (SH9H‐2023‐A849‐SB) and performed in compliance with institutional ethical guidelines. Genotyping was conducted using mouse tissue direct PCR kit (Yeasen, Shanghai) and primers are listed in Table S1. To induce acute colitis, 8‐week‐old Setd2^f/f^ and Setd2^vil‐ko^ littermates were fed 2% (w/v) dfragments per kilobase of transcript per million mapped readsextran sulfate sodium salt (DSS) (36‒50 kDa, MP Biomedals) for 5 consecutive days, followed by 5 days of normal drinking water. Daily assessments were performed to evaluate body weight change, diarrhoea and rectal bleeding in mice. Disease activity index (DAI) was calculated as: DAI = (weight loss scores + stool consistency scores + bleeding scores)/3.^22^ Animals exhibiting a body weight loss of greater than 20% over a 7‐day period will be humanely euthanised and recorded as study mortalities. Mice with antibiotics (ABX) treatment were administered with a cocktail of vancomycin (.5 g/L, HY‐B0671, MedChemExpress), ampicillin (1 g/L, HY‐B0522, MedChemExpress), neomycin (1 g/L, HY‐B0470, MedChemExpress) and metronidazole (1 g/L, HY‐B0318, MedChemExpress) 7 days before being exposed to DSS or undergoing faecal microbiome transplantation (FMT). Colon tissues were harvested on day 10 post‐treatment for downstream experimental applications

### 2.2 Haematoxylin and eosin staining and histopathological scoring

Following a 24 h fixation in 10% formalin, intestinal tissues were dehydrated and sliced into 4 µm sections. Routine haematoxylin and eosin (H&E) staining was subsequently performed using an H&E staining kit (G1076, Servicebio). Previously described pathological scoring system^23^ was used to evaluate colitis severity. Histological assessment was based on four parameters: loss of crypts (0–4: none, basal one‐third, basal two‐thirds, surface epithelium only intact, or complete loss of crypts and epithelium), extent of injury (0–3: none, mild, moderate, severe), mucosal damage (0–3: none, mucosal, mucosal–submucosal, transmural) and inflammatory cell infiltration (0–4: none, 1%–25%, 26%–50%, 51%–75%, >75%). The total histological score, ranging from 0 to 14, was the sum of these four subscores.

### 2.3 Isolation of IECs

Following the removal of faecal contents, colons were incised longitudinally and subsequently sectioned into 3‒5 mm pieces in phosphate buffered saline (PBS). Colon pieces were placed in 10 mL of 8 mM EDTA solution and incubated for 20 min at 37°C. Following incubation, the EDTA solution was aspirated, and resuspended tissue in PBS. Cell suspension was vigorously shaken for 1 min to dissociate IECs. The procedure was repeated, and cell suspensions from both rounds were pooled. The combined cell suspension was filtered through a 100 µm cell strainer and centrifuged at 2000 rpm for 2 min at 4°C to obtain IECs.

### 2.4 RNA extraction and quantitative PCR

Total RNA was extracted using TRIzol reagent (15596026CN, Invitrogen) and reverse‐transcribed into cDNA with the ProFlex PCR system (Applied Biosystems). Quantitative PCR (qPCR) was performed on a QuantStudio 6 Pro Real‐Time PCR System (Applied Biosystems), and mRNA expression levels were calculated using the comparative ΔΔCT method, with GAPDH as the reference housekeeping gene. Primers used in this study are listed in Table S2.

### 2.5 Protein lysis and immunoblotting

Total proteins were extracted using lysate. Bicinchoninic Acid kit (Thermo Fisher Scientific) was used to detect protein concentration. Primary antibodies used for immunoblotting were as follows: anti‐REG3A (Abmart, Cat# PS02575, 1:1000); anti‐REG3B (R&D Systems, Cat# AF5110, 1:1000); anti‐REG3G (Abclonal, Cat# A2146, 1:1000); and β‐actin (Proteintech, Cat# 11837‐1‐AP, 1:5000). Quantification analysis of immunoblot was performed using ImageJ software (NIH).

### 2.6 Immunofluorescence staining and quantification

Paraffin‐embedded and formalin‐fixed colon tissues of Setd2^f/f^ and Setd2^vil‐ko^ mice were used for immunofluorescence staining. Primary antibodies were as follows: anti‐REG3A (Abmart, Cat# PS02575, 1:200); anti‐REG3B (R&D Systems, Cat# AF5110, 1:50); anti‐REG3G (Abclonal Cat# A2146, 1:200); and anti‐EPCAM/CD326 (Proteintech, Cat# 21050‐1‐AP, 1:3000). Immunofluorescence staining for REG3 proteins and EPCAM was performed according to the instructions of TSA kit (Runner Bio). Briefly, tissue slides were dewaxed and hydrated and then treated with 2% hydrogen peroxide. Following antigen retrieval with EDTA and blocking with 3% goat serum, sections were incubated with REG3 primary antibodies for 2 h at room temperature, then with goat anti‐rabbit IgG H&L (horseradish peroxidase) secondary antibody (ab205718, Abcam; 1:2000) or anti‐sheep IgG H&L (horseradish peroxidase) secondary antibody (313‐035‐003, Jackson; 1:200). The above steps were repeated to perform EPCAM antibody staining. Nuclei were counterstained by DAPI. Quantification of the mean fluorescence intensity in positive proteins was performed using ImageJ software (NIH).

### 2.7 RNA sequencing and data analysis

Colonic IECs were isolated from DSS‐treated (5 days) Setd2^f/f^ and Setd2^vil‐ko^ mice according to the methods described above. RNA sequencing (RNA‐seq) libraries were constructed using the NEBNext Ultra Directional RNA Library Prep Kit (New England Biolabs). Sequencing was performed on an Illumina platform with paired‐end 2 × 150 bp configuration. Raw sequencing data underwent quality filtering to generate high‐quality reads, with adapter sequences, reads shorter than 35 bp, and low‐quality bases removed using Cutadapt (v1.18) and Trimmomatic (v0.35). Subsequent quality assessment was performed via FastQC (v0.11.9), followed by alignment to the mouse reference genome (mm10) using HISAT2 (v2.1.0). Gene expression quantification was performed using StringTie (v1.3.4d) to calculate fragments per kilobase of transcript per million mapped reads (FPKM) values. Differential expressed gene (DEG) analysis was conducted with edgeR (v3.24.2), identifying genes with adjusted *p*‐value < .05 and |log_2_FC| ≥ 1 for downstream analysis. Functional annotation for Gene Ontology (GO) terms and Kyoto Encyclopedia of Genes and Genomes (KEGG) pathways was executed using the ClusterProfiler R package (v4.0.0), with enrichment analysis performed via the same tool. Gene set enrichment analysis (GSEA) was performed using the GSEA software (http://www.broadinstitute.org/gsea).

### 2.8 HT‐29 cell culture and treatment

HT‐29 cell line was purchased from the American Type Culture Collection (ATCC). HT‐29 cells were maintained in RPMI‐1640 medium (Gibco) containing 10% fetal bovine serum (FBS) and 1% penicillin–streptomycin under standard culture conditions. To generate SETD2‐konckout HT‐29 cells, sgRNA targeting SETD2 was designed (‘AAGTATCTAGCTTCTAACCA’)^24^ and cloned into a lentiviral vector (lentiCRISPRv2). Cells were transfected using EZ transfection regime according to the manufacturer's instructions. To build the in vitro IBD model, the transfected HT‐29 cells were subjected to lipopolysaccharide (LPS) stimulation. Briefly, cells were initially starved in serum‐free medium for 2 h to synchronise cell cycles. Subsequently, the culture medium was replenished with RPMI‐1640 medium supplemented with 5% FBS containing 20 µg/mL of LPS (Cat# L2880, Sigma). Following a 6 h incubation under inflammatory condition, cells were harvested for downstream analysis.

### 2.9 Assay for transposase‐accessible chromatin with high‐throughput sequencing and data analysis

Samples were prepared using the Qiagen MinElute Kit. Assay for transposase‐accessible chromatin with high‐throughput sequencing (ATAC‐seq) library amplification was performed with PCR primers, 5× Tn5 amplification buffer, 10× PCR purification buffer and Tris‐Acetate‐EDTA buffer (all Vazyme). Resulting libraries were sequenced on Illumina platform by Genefund Biotech (Shanghai, China). After quality control, clean reads were aligned to the mouse reference genome (assembly GRCm38) via Bowtie2 (v2.3.4.1). Duplicate reads were removed using Picard MarkDuplicates. Peak calling was performed and peak annotations to gene features were conducted using ChIPseeker R package. Functional enrichment analysis (GO and KEGG) was performed with ClusterProfiler (v3.4.4). Differential peak analysis was executed using The DiffBind with a significance threshold of *p* < .05.

### 2.10 Cleavage under targets and tagmentation analyses

Cleavage under targets and tagmentation (CUT&Tag) Assay Kit for Illumina (Vazyme, TD903) was used to construct libraries. In brief, IECs were isolated from DSS‐treated Setd2^f/f^ and Setd2^vil‐ko^ mice. Then, nuclei harvested from fresh IECs were incubated with ConA beads and then separately incubated with primary antibodies targeting H3K36me3 (Abcam, Cat# ab282572) and H3K4me3 (Abcam, Cat# ab8580), with a non‐specific IgG serving as the negative control, followed by secondary antibody incubation. Tagmentation was performed by introducing the pA‐Tn5 adapter complex. After subsequent washing, fragment release and proteinase K digestion, the purified DNA fragments were recovered. Library amplification was achieved via 12 cycles of PCR. Finally, sequencing was conducted on Illumina NovaSeq 6000 platform by Genefund Biotech (Shanghi, China). Bioinformatics analysis was performed by Genefund Biotech (Shanghai, China).

### 2.11 16S rRNA sequencing and data analysis

Faecal samples were collected from water or DSS‐treated (5 days) Setd2^f/f^ and Setd2^vil‐ko^ mice. Genomic DNA was extracted from faecal samples using the FastPure Stool DNA Isolation Kit (MJYH). Sequencing was performed on Illumina NextSeq 2000 platform by Majorbio company.

Raw sequencing reads were demultiplexed using an in‐house Perl script, then quality‐controlled with fastp (v0.19.6) and merged with FLASH (v1.2.7). High‐quality sequences were clustered into operational taxonomic units (OTUs) at a 97% similarity threshold using UPARSE (v7.1), with the most abundant sequence in each OTU selected as its representative. To normalise sequencing depth, samples were rarefied to 20 000 reads. Representative OTU sequences were then annotated against the SILVA database (v138) using the RDP Classifier (v2.2) with a .7 confidence threshold.

### 2.12 Non‐targeted metabolomics analysis

Faecal samples were collected from water or DSS‐treated (5 days) Setd2^f/f^ and Setd2^vil‐ko^ mice. Metabolite profiling was performed on a UHPLC‐Orbitrap Exploris 480 system (Thermo Fisher Scientific). Raw data processing was executed using Progenesis QI software (Waters Corporation) to generate a matrix. Metabolite identification was achieved by searching against HMDB, Metlin, and the proprietary Majorbio Database. The processed dataset was analyzed on Majorbio Cloud platform.

### 2.13 Faecal microbiome transplantation

The fresh faecal feces were collected from healthy wild‐type donor mice, homogenised in sterile PBS at a concentration of 100 mg/mL, vortexed for 3 min, and then centrifuged at 1500 rpm for 3 min to remove insoluble debris. The resulting supernatant was immediately administered via oral gavage (100 µL per mouse) to minimise air exposure. Control mice received an equivalent volume of sterile PBS (100 µL) by oral gavage. FMT was performed once daily for 14 consecutive days, after which all mice were subjected to DSS administration.

### 2.14 Public datasets acquisition and processing

To evaluate the clinical relevance of SETD2 and REG3, public bulk RNA‐seq datasets of patients with IBD were retrieved from the Gene Expression Omnibus database. Specifically, normalised expression matrices of 125 IBD samples from GSE207022 (https://www.ncbi.nlm.nih.gov/geo/query/acc.cgi?acc =gse207022) and 26 IBD samples from GSE9452 (https://www.ncbi.nlm.nih.gov/geo/query/acc.cgi?acc=gse9452). Following quality control and quantile normalisation, Pearson correlation analysis was performed to assess the expression correlation between SETD2 and REG3 genes.

To further resolve this correlation at single‐cell resolution, we reanalysed our previously published single‐cell scRNA‐seq dataset of 17 IBD samples (HRA007064, https://ngdc.cncb.ac.cn/gsa‐human/browse/HRA007064).^25^ Following standard quality control filtering (including cell viability, gene counts per cell and mitochondrial molecule percentages) and data normalisation via the Seurat package. Pearson correlation analysis between SETD2 and REG3 expression was specifically conducted within the IECs with the expression of both genes exceeded the lower quartile of their respective non‐zero expression values.

### 2.15 Statistical analysis

All experiments were replicated in a minimum of three independent experimental runs. Data are presented as the mean ± SEM. Normally distributed continuous data were evaluated using Student's *t*‐test, whereas the Mann‒Whitney *U* test was applied for non‐normally distributed data. Pearson correlation analysis was executed to assess variables relationship. All statistical computing was conducted via GraphPad Prism (v9.0) and R software (v4.1.0). A two‐sided *p *< .05 was considered statistically significant.

## 3 RESULTS

### 3.1 SETD2 deficiency in IECs aggravates colitis and promotes antibacterial genes expression

**FIGURE 1:**
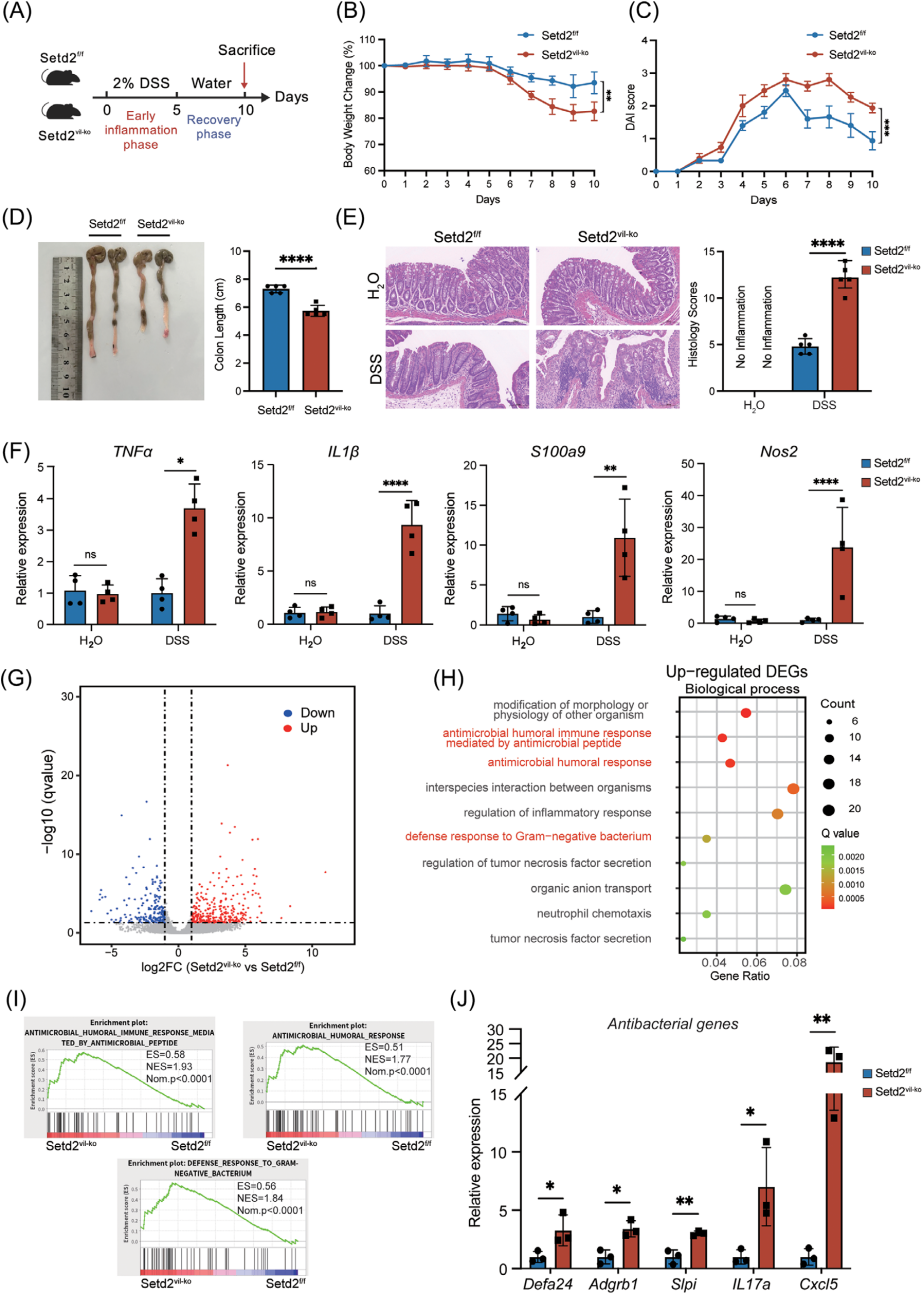
SET domain containing 2 (SETD2) deficiency in intestinal epithelial cells (IECs) aggravates colitis and promotes antibacterial genes expression. (A) Schematic illustration of the DSS‐induced colitis protocol. (B) Daily body weight changes in mice. *n* = 5 mice per group. (C) Disease activity index (DAI) reflecting the inflammatory status. (D) Representative images of colon shortening and statistical analysis in DSS‐treated mice. (E) Haematoxylin and eosin (H&E) staining of colon tissues from water‐treated and DSS‐treated mice and corresponding quantification of histology scores. (F) Relative mRNA expression levels of *TNFα*, *IL1β*, *S100a9* and *Nos2* determined by real‐time qPCR in water‐treated and DSS‐treated mice. (G) Differentially expressed genes (DEGs) between Setd2^f/f^ (*n* = 3) and Setd2^vil‐ko^ (*n* = 3) mice. Blue and red bubbles represent significant downregulated and upregulated DEGs in Setd2^vil‐ko^, respectively. (H) Gene Ontology (GO) analysis of upregulated DEGs in Setd2^vil‐ko^ by RNA sequencing (RNA‐seq). Deeper red colour indicates a lower *q*‐value and greater enrichment significance. Larger bubbles denote a higher number of enriched genes. All data are presented as mean ± standard deviation (SD). (I) Gene set enrichment analysis (GSEA) plot illustrating the enrichment patterns of antimicrobial biological processes. (J) Relative mRNA expression levels of *Defa24, Adgrb1*, *Slpi*, *IL17a* and *Cxcl5* determined by real‐time qPCR in Setd2^f/f^ (*n* = 3) and Setd2^vil‐ko^ (*n* = 3) mice. For body weight curve and DAI curve, *p*‐values were calculated at day 10. *p*‐Values were calculated using two‐tailed Student's *t*‐test. ^*^ *p* < .05; ^**^ *p* < .01; ^***^ *p* < .001; ^****^ *p* < .0001.

SETD2 is a critical epigenetic regulator and H3K36 methyltransferase enzyme. The absence or dysfunction of SETD2 has been implicated in the pathogenesis of various diseases.^8^, ^9^, ^10^ To determine the functional role and physiological mechanism of SETD2 in intestinal homeostasis and inflammation, we generated IEC‐specific SETD2 knockout mice (Setd2^vil‐ko^) by crossing Setd2^f/f^ mice with villin‐cre mice (Figure S1). Subsequently, we treated Setd2^vil‐ko^ mice and Setd2^f/f^ littermates with 2% DSS solution to induce colitis (Figure 1A). Setd2^vil‐ko^ mice exhibited significantly exacerbated clinical manifestations of colitis in the model‐building process. A more rapid and substantial body weight loss was observed in Setd2^vil‐ko^ mice, and concurrent with higher DAI scores (Figure 1B, C). At the time of sacrifice, Setd2^vil‐ko^ mice displayed significantly shorter colon lengths compared to controls (Figure 1D). Histopathological analysis showed severe crypt disruption, epithelial barrier damage and submucosal inflammatory infiltration in Setd2^vil‐ko^ mice, with higher histological scores confirming exacerbated intestinal injury (Figure 1E). In addition, qPCR analysis revealed an elevated expression of inflammatory markers (*TNFα*, *IL1β*, *S100a9* and *Nos2*) in the IECs isolated from Setd2^vil‐ko^ mice (Figure 1F). To exclude baseline phenotypic variations, we compared the inflammatory profiles between water‐treated Setd2^f/f^ and Setd2^vil‐ko^ mice, finding no significant differences (Figures 1E, F and S2).

To elucidate the molecular mechanisms underlying the aggravated phenotype, we performed RNA‐seq on colonic IECs isolated from Setd2^f/f^ and Setd2^vil‐ko^ mice after DSS treatment. DEG analysis revealed a large number of altered transcripts, including 303 upregulated genes and 188 downregulated genes in Setd2^vil‐ko^ mice (Figure 1G). GO enrichment analysis of DEGs revealed significant activation of antimicrobial pathways, with the upregulated genes predominantly enriched in biological processes, including ‘antimicrobial humoral immune response mediated by antimicrobial peptides’, ‘antimicrobial humoral response’ and ‘defense response to Gram‐negative bacteria’ (Figure 1H). GSEA further confirmed upregulation of antimicrobial biological processes in Setd2^vil‐ko^ mice (Figure 5I). Real‐time qPCR validated an increase in the mRNA levels for antibacterial genes, including *Defa24*, *Adgrb1*, *Slpi*, *IL17a* and *Cxcl5* (Figure 1J). IECs form a physical and biochemical barrier separating host tissues from commensal microbiota while maintaining intestinal homeostasis through microbial signal sensing.^26^ These findings indicated that the loss of SETD2 triggered dysregulation and hyperactivation of antimicrobial responses, potentially driving the increased susceptibility and severity of colitis.

### 3.2 Dramatic upregulation of REG3 family genes in SETD2‐deficient intestinal epithelium

**FIGURE 2:**
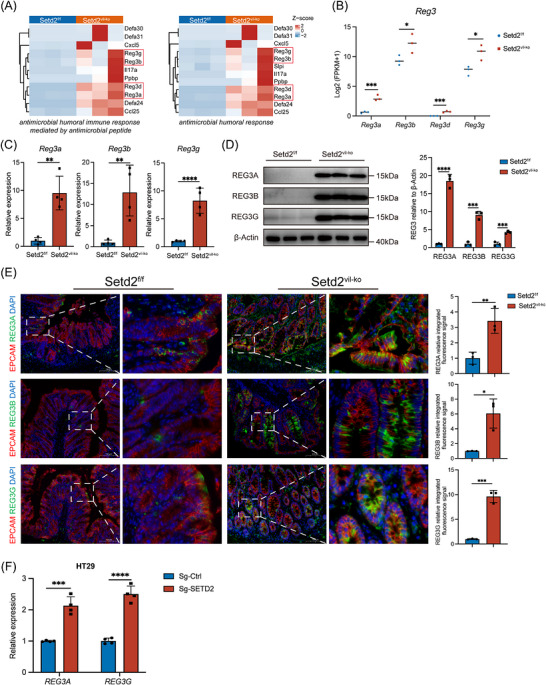
Significant upregulation of REG3 in Setd2^vil‐ko^ colonic mucosa. (A) Heatmap illustrating the genes involved in the biological processes of ‘antimicrobial humoural immune response mediated by antimicrobial peptide’ (left) and ‘antimicrobial humoural response’ (right). (B) Expression levels of *Reg3a*, *Reg3b*, *Reg3d* and *Reg3g* in the intestinal epithelial cells (IECs) of DSS‐treated Setd2^f/f^ and Setd2^vil‐ko^ mice detected by RNA sequencing (RNA‐seq). Gene expression levels underwent log2 + 1 transformation. (C) Relative mRNA expression levels of *Reg3a*, *Reg3b* and *Reg3g* determined by real‐time qPCR in Setd2^f/f^ (*n* = 4) and Setd2^vil‐ko^ (*n* = 4) mice. (D) Immunoblot showing the expression of REG3A, REG3B and REG3G in IECs isolated from Setd2^f/f^ (*n* = 3) and Setd2^vil‐ko^ (*n* = 3) mice (left). Quantitative analysis of immunoblot with ImageJ (right). β‐Actin was used as a control. (E) Representative images of immunofluorescence staining for EPCAM (red) and REG3 proteins (green) in colon tissues from Setd2^f/f^ (*n* = 3) and Setd2^vil‐ko^ (*n* = 3) mice (left). Quantitative analysis of fluorescence signal with ImageJ (right). (F) Relative mRNA expression levels of REG3A, REG3G in HT‐29 cells. HT‐29 cells were transfected with either the control vector or sgSETD2, and were stimulated with lipopolysaccharide (LPS). All the data are presented as mean ± standard deviation (SD). *p*‐Values were calculated using two‐tailed Student's *t*‐test. ^*^ *p* < .05; ^**^ *p* < .01; ^***^ *p* < .001; ^****^ *p* < .0001.

Further functional analysis of the upregulated DEGs focused on antimicrobial pathways. Surprisingly, we noticed that host defense genes Reg3 (*Reg3a*, *Reg3b*, *Reg3d* and *Reg3g*) exhibiting pronounced overexpression in the IECs isolated from DSS‐induced Setd2^vil‐ko^ mice (Figure 2A). REG3 lectins encoded by Reg3 genes are among the most important AMPs produced within the gut mucosa, playing a fundamental role in maintaining the spatial segregation and homeostasis between the gut microbiota and host tissue.^27^ In humans, the Reg3 family members include *REG3A* (*REG3α*) and *REG3G* (*REG3γ*). In mice, the homologous Reg3 family comprises *Reg3a (Reg3α)*, *Reg3b (Reg3β)*, *Reg3g (Reg3γ)* and *Reg3d (Reg3δ)*.^28^ Given the minimal expression of *Reg3d* in the IECs (Figure 2B), our investigation focused on the expression changes of *Reg3a*, *Reg3b* and *Reg3g*. Consistently, qPCR analysis revealed that mRNA levels of *Reg3a*, *Reg3b* and *Reg3g* were significantly elevated in the IECs of Setd2^vil‐ko^ mice compared to Setd2^f/f^ littermates following DSS induction (Figure 2C). Immunoblotting further confirmed the increased expression of REG3A, REG3B and REG3G at protein levels (Figure 2D). Additionally, increased expression of REG3 proteins was predominantly evident in the colonic epithelium as displayed by immunofluorescence staining (Figures 2E and S3). Consistently, the in vitro IBD model established using HT‐29 cells transfected with either the control vector or sgSETD2 confirmed that SETD2 deficiency leads to a robust upregulation of REG3 expressions (Figures 2F and S4). Collectively, these robust data demonstrated that SETD2 deficiency resulted in the dysregulated overexpression of the Reg3 genes. We hypothesise that SETD2 functions as a previously unrecognised epigenetic regulator of Reg3 genes expression.

### 3.3 REG3 upregulation induced by SETD2 deficiency involves enhanced chromatin accessibility and H3K4me3 activation

**FIGURE 3:**
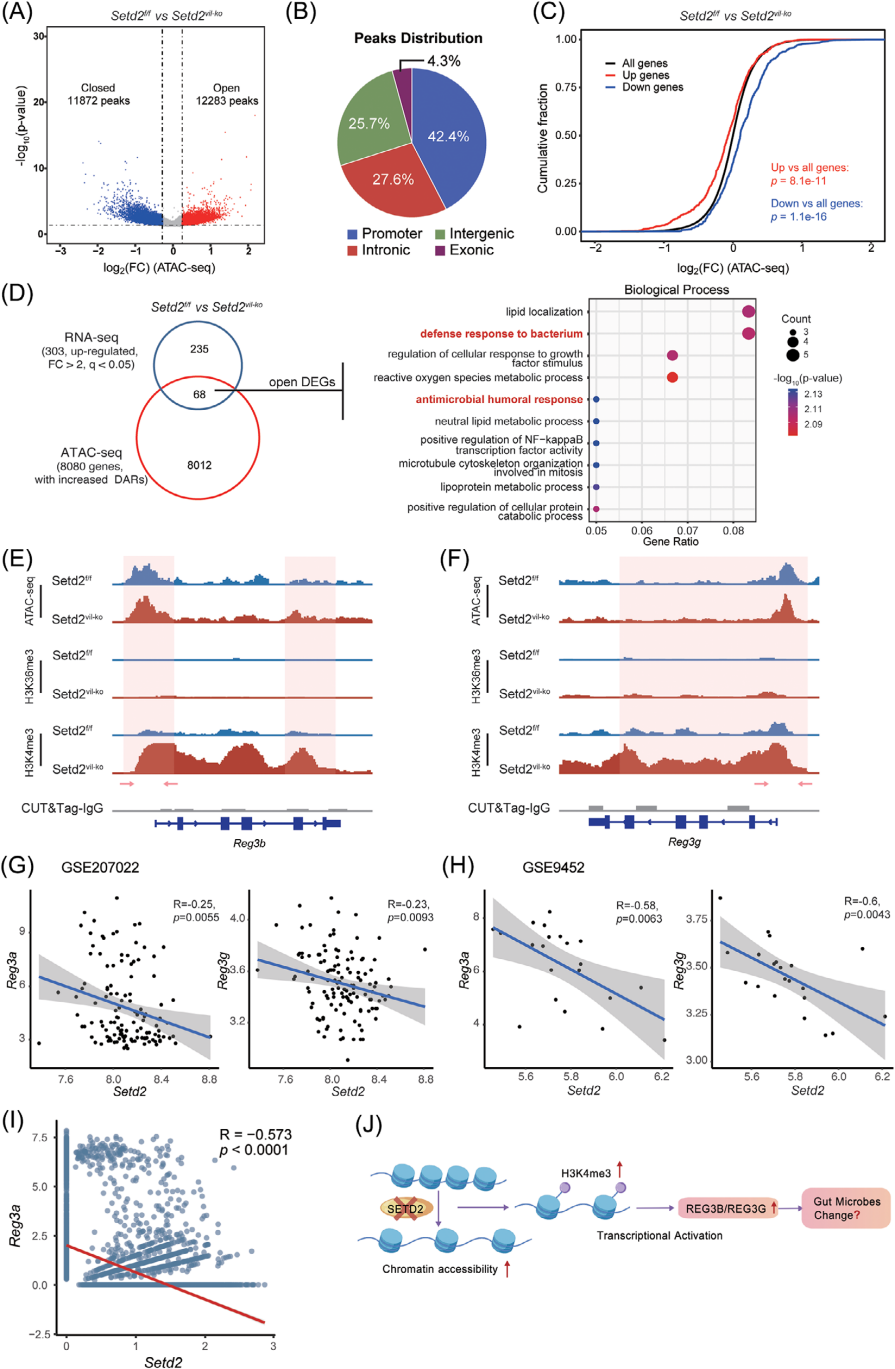
REG3 upregulation induced by SET domain containing 2 (SETD2) deficiency involves enhanced chromatin accessibility and H3K4me3 activation. (A) Volcano plots of assay for transposase‐accessible chromatin with high‐throughput sequencing (ATAC‐seq) differential peaks. The number of peaks with significant changes (*p *< .05 and |log_2_FC| > .263) upon Setd2 deletion is shown. (B) Pie chart illustrating the proportion of differentially accessible ATAC‐seq peaks located in promoter, intronic, intergenic and exonic regions. (C) Cumulative distribution of chromatin accessibility changes associated with significantly upregulated (red) or downregulated (blue) genes (*q* < .05 and |log_2_FC| > 1) in Setd2^vil‐ko^ mice compared with Setd2^f/f^ controls. *p*‐Values were calculated using a two‐sided Kolmogorov‒Smirnov test comparing peaks associated with differentially expressed genes (DEGs) to all genes. (D) Integrated analysis of upregulated DEGs from RNA sequencing (RNA‐seq) and genes with increased differentially accessible regions (DARs) from ATAC‐seq, termed as open DEGs (left). Gene Ontology (GO) analysis of the 68 open DEGs identified antimicrobial‐related biological processes (right). (E and F) Representative ATAC‐seq signals, H3K36me3 and H3K4me3 signals (cleavage under targets and tagmentation sequencing [CUT&Tag‐seq]) at the *Reg3b* (E) and *Reg3g* (F) gene loci in intestinal epithelial cells (IECs) from DSS‐treated (5 days) Setd2^f/f^ and Setd2^vil‐ko^ mice. Pink, differentially accessible regions. Arrows indicate signals in promotor regions. (G and H) Scatter plot showing the correlation between expression levels of *Setd2*, *Reg3a* and *Reg3g* in inflamed Crohn's disease (CD) (G) and ulcerative colitis (UC) colon (H) from Gene Expression Omnibus (GEO) microarray datasets GSE207022 and GSE9452. Pearson's *R*‐value and *p*‐value for the correlation coefficient are shown on the upper right side. (I) Correlation analysis of the expression levels of *Setd2* and *Reg3a* in IECs from our former single‐cell RNA‐seq dataset (HRA007064). Pearson's *R*‐value and *p*‐value were determined across cells expressing both genes at levels above their respective 25th percentiles of non‐zero expression values. (J) Schematic illustration of the hypothesis that SETD2 deficiency alters expressions of REG3 family members.

Given that SETD2 is a pivotal histone methyltransferase and chromatin accessibility is intimately linked to transcriptional regulation,^29^, ^30^ we hypothesised that SETD2 deficiency might alter the chromatin structure at Reg3 genes. ATAC‐seq was performed on the IECs from Setd2^f/f^ and Setd2^vil‐ko^ mice to assess the changes of chromatin accessibility. SETD2 deficiency in IECs induced significant genome‐wide chromatin accessibility changes with 12 283 open peaks and 11 872 closed peaks (Figure 3A). Among the differentially accessible ATAC‐seq peaks (FDR < .05), 42.4% were found at promotors, 27.6% at introns, 25.7% at exons and 4.3% at intergenic regions (Figure 3B). Integrated analysis of RNA‐seq and ATAC‐seq data revealed the correlation between chromatin accessibility and transcriptional expressions induced by SETD2 deficiency. The upregulated genes in the IECs from Setd2^vil‐ko^ mice exhibited more open chromatin, while the downregulated genes showed more closed chromatin (Figure 3C). Notably, SETD2 deficiency‐induced genes with increased differentially accessible regions (DARs) (termed as open DEGs) were functionally involved in antimicrobial biological processes, such as ‘defense response to bacterium’ and ‘antimicrobial humoral response’ (Figure 3D). Consistent with the elevated mRNA levels observed in RNA‐seq, ATAC‐seq tracks revealed significantly increased chromatin accessibility at both promotor and gene body of *Reg3b* and *Reg3g* upon SETD2 deficiency (Figure 3E,F). Previous studies reported that that impaired H3K36me3 deposition might drive the aberrant over‐establishment of H3K4me3.^31^, ^32^, ^33^, ^34^ To investigate the underlying epigenetic landscape regulating chromatin accessibility at Reg3 loci, we next conducted Cut&Tag‐seq targeting both H3K36me3 and H3K4me3. On a genome‐wide scale, the global signal of H3K36me3 was notably diminished in Setd2^vil‐ko^ mice, while an overall increase in H3K4me3 was observed (Figure S5). Intriguingly, no discernible H3K36me3 peaks were detected at the Reg3 loci in either group. In contract, the signal for transcription‐activating mark H3K4me3 was significantly elevated at the promoters of *Reg3b* and *Reg3g* in Setd2^vil‐ko^ mice, suggesting an indirect epigenetic mechanism whereby SETD2 deficiency promotes Reg3 expression via local H3K4me3‐mediated promoter activation rather than conventional H3K36me3 modification (Figure 3E,F).

Additionally, analysis of the public datasets from patients with IBD indicated the negative correlation between the mRNA levels of *Setd2*, *Reg3a* and *Reg3g*, respectively (Figure 3G,H). To further validate our findings in clinical samples, we reanalyzed our previously published single‐cell RNA sequencing (scRNA‐seq) dataset, which characterised the single‐cell transcription atlas of patients with CD.^25^ Our analysis specifically focused on the expression of *Setd2* and Reg3 genes within IECs. To mitigate the technical dropout effect inherent in scRNA‐seq data, correlation analysis was restricted to cells where the expression of both genes exceeded the lower quartile of their respective non‐zero expression values. Notably, a significant negative correlation between *Setd2* and *Reg3a* expression levels was revealed (Figure 3I, *R *= ‒.573, *p* < .0001). These findings further demonstrated that SETD2 negatively regulates REG3 expressions. Collectively, we propose that loss of the epigenetic regulator SETD2 triggers an open chromatin landscape and local H3K4me3 hyper‐enrichment at Reg3 genes, thereby driving transcriptional upregulation. The dysregulated overexpression of Reg3 genes may not entirely protective but instead might exacerbate colitis by disrupting microbial homeostasis (Figure 3J).

### 3.4 Gut microbiota dysbiosis is indispensable for SETD2 deficiency‐induced colitis exacerbation

**FIGURE 4:**
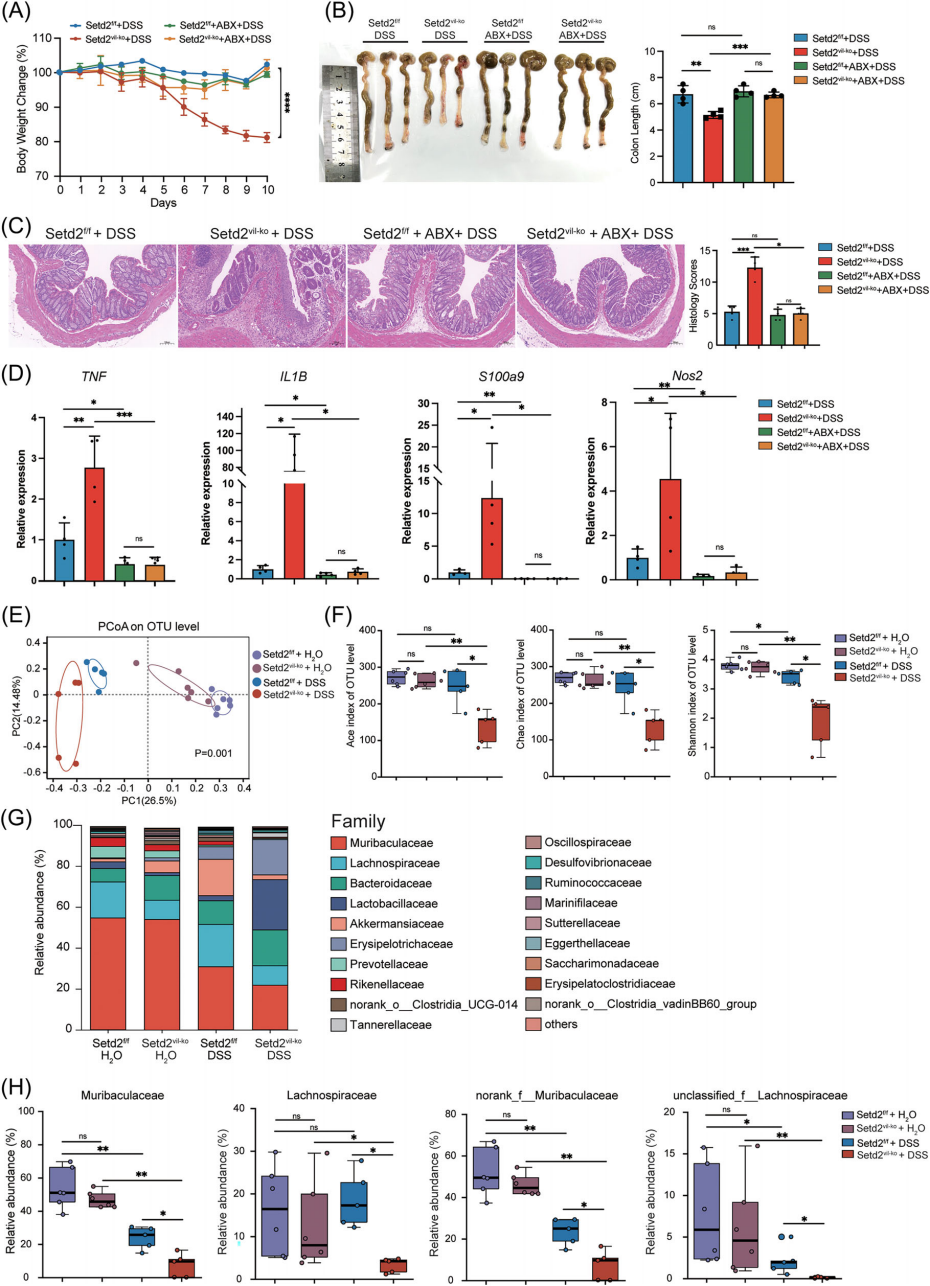
Gut microbiota dysbiosis is indispensable for SET domain containing 2 (SETD2) deficiency‐induced colitis exacerbation. (A) Daily body weight changes in mice. *n* = 4 mice per group. (B) Representative images of colon shortening and statistical analysis. (C) Haematoxylin and eosin (H&E) staining of colon tissues collected on day 10 and corresponding quantification of histology scores. (D) Relative mRNA expression levels of *TNFα*, *IL1β*, *S100a9* and *Nos2* determined by real‐time qPCR in antibiotics (ABX)‐treated or untreated Setd2^f/f^ and Setd2^vil‐ko^ mice. (E) Principal coordinates analysis (PCoA) atlas of the faecal microbial communities. (F) Alpha diversity analysis shown by the Ace index, Chao index and Shannon index. (G) Relative abundance of faecal microbial communities at the family level in water‐ and DSS‐treated Setd2^f/f^ and Setd2^vil‐ko^ mice. (H) Relative abundance of indicated gut microbiota in water‐ and DSS‐treated Setd2^f/f^ and Setd2^vil‐ko^ mice. All the data are presented as mean ± standard deviation (SD). For body weight curve, *p*‐values were calculated between ABX‐untreated and untreated Setd2^vil‐ko^ mice at day 10. *p*‐Values for 16s rRNA‐seq were calculated using Mann‒Whitney *U* test. *p*‐Values for others were calculated using two‐tailed Student's *t*‐test. ^*^ *p* < .05; ^**^ *p* < .01; ^***^ *p* < .001; ^****^ *p* < .0001.

As a critical IEC‐derived AMPs, REG3 lectins reinforce intestinal barrier function and are critical for the maintenance of microbiota homeostasis.^28^ However, overproduction of REG3 lectins can be detrimental by reducing protective gut microbiota.^35^, ^36^ Given the constitutive upregulation of *Reg3* in Setd2^vil‐ko^ mice, we hypothesised that SETD2 deficiency might reshape the gut microbiota compositions through enhanced REG3‐mediated antimicrobial activity. To investigate if alterations in gut microbiota were involved in the exacerbated intestinal inflammation, we treated both Setd2^f/f^ and Setd2^vil‐ko^ mice with ABX followed by inducing colitis with DSS (Figure S6A). Notably, the increased susceptibility to DSS‐induced colitis typically observed in Setd2^vil‐ko^ mice was effectively abolished upon the elimination of the gut microbiota. After undergoing ABX treatment, body weight loss and DAI scores of Setd2^vil‐ko^ mice were indistinguishable from those of the control group (Figures 4A and S6B), contrasting sharply with the severe phenotype observed in Setd2^vil‐ko^ mice without ABX treatment. Consistent with the clinical manifestations, colon lengths in the ABX‐treated Setd2^vil‐ko^ mice were comparable to those of the control littermates, showing no significant shortening (Figure 4B). Histological analysis and qPCR analysis of proinflammatory mediators further confirmed the rescue of the exacerbated colitis phenotype (Figure 4C,D). These data demonstrate that the severe colitis phenotype observed in Setd2^vil‐ko^ mice is dependent on the presence of gut bacteria.

We next characterised the alterations in gut microbiota composition using 16S rRNA sequencing in both Setd2^f/f^ and Setd2^vil‐ko^ mice treated with either water or DSS. We first evaluated the overall microbial community structure. Principal coordinate analysis (PCoA) based on bray_crutis distances revealed a distinct clustering separation between the four groups, indicating a substantial shift in the overall microbiota composition following SETD2 deletion and inflammation (Figure 4E). Furthermore, we assessed alpha diversity to determine microbial richness and evenness. While no significant differences in alpha diversity metrics were observed between Setd2^f/f^ and Setd2^vil‐ko^ mice under homeostatic conditions (water‐treated groups), DSS challenge induced a profound divergence. Specifically, under inflammatory conditions, Setd2^vil‐ko^ mice exhibited a significant reduction in microbial within‐community diversity, as evidenced by decreased Ace, Chao and Shannon indexes compared to littermate controls (Figure 4F). The overall evaluations of the relative abundance of bacteria on family level in the four groups were displayed in Figure 4G. The linear discriminant analysis effect size (LEfSe) approach based on linear discriminant analysis (LDA) was used to identify differentially abundant features across groups in high‐dimensional data (Figure S6C,D). Under homeostatic conditions, the overall microbial structures were similar between the two genotypes (Figure 4G). Upon DSS challenge, the reduction of both *Muribaculaceae* and *Lachnospiraceae* was much more severe in Setd2^vil‐ko^ mice, which was consistent at the genus level, affecting representative taxa from both families (Figure 4H). The more pronounced depletion of gut commensals in DSS‐treated Setd2^vil‐ko^ mice with high REG3 expression, is consistent with the established role of REG3 lectins in restricting bacterial translocation and regulating host‒microbiota interactions.^37^, ^38^, ^39^

### 3.5 SETD2 deficiency in IECs alters faecal metabolites during colitis

**FIGURE 5:**
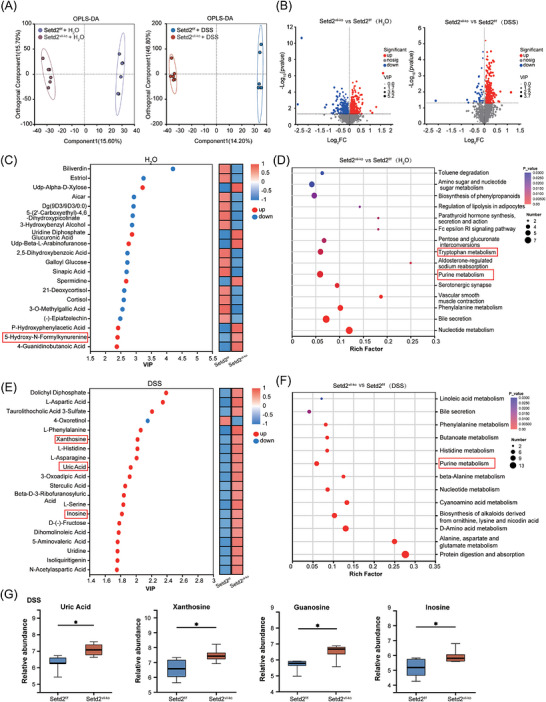
SET domain containing 2 (SETD2) deficiency in intestinal epithelial cells (IECs) alters faecal metabolites during colitis. (A) Orthogonal partial least square discriminant analysis (OPLS‐DA) plot of the faecal metabolites in water‐treated Setd2^f/f^ and Setd2^vil‐ko^ mice (left, R2Y = .996, Q2 = .606) and DSS‐treated Setd2^f/f^ and Setd2^vil‐ko^ mice (right, R2Y = .999, Q2 = .835); *n* = 6 mice per group. (B) Volcano plot of differential metabolites (*p* < .05, variable importance in projection [VIP] > 1 and fold change > 1). (C and D) VIP value diagram (C) and KEGG enrichment analysis (D) between water‐treated Setd2^f/f^ and Setd2^vil‐ko^ mice. (E and F) VIP value diagram (E) and KEGG enrichment analysis (F) between DSS‐treated Setd2^f/f^ and Setd2^vil‐ko^ mice. (G) Comparison of the relative abundance of purine metabolites in DSS‐treated mice. All the data are presented as mean ± standard deviation (SD). *p*‐Values were calculated using two‐tailed Student's *t*‐test. ^*^ *p* < .05; ^**^ *p* < .01; ^***^ *p* < .001; ^****^ *p* < .0001.

To further explore how SETD2 deficiency in IECs affects colitis, we performed untargeted metabolomic analysis in stool samples from Setd2^f/f^ and Setd2^vil‐ko^ mice treated with either water or DSS. The orthogonal partial least square discriminant analysis (OPLS‐DA) revealed clear differences in metabolic profiles between Setd2^f/f^ and Setd2^vil‐ko^ mice, under both homeostatic and inflammatory conditions (Figure 5A). The permutation test analysis (200 times) showed that the models were not overfitted, and the regression line exhibiting an upward trend from left to right (Figure S7A,B).

We next analysed the differentially abundant metabolites under water and DSS conditions separately (Figure 5B). We observed a baseline metabolic shift in Setd2^vil‐ko^ mice characterised primarily by the downregulation of specific metabolites, alongside a notable upregulation of intermediates within the tryptophan metabolism pathway (Figures 5C,D and S7C,E). In sharp contrast, following DSS challenge, we found a considerable number of metabolites exhibiting significantly elevated expression levels and abnormal accumulation in the Setd2^vil‐ko^ mice (Figure 5D). Metabolite clustering analysis and variable importance in projection (VIP) scoring indicated the abnormal accumulation of purine, amino acid and lipid metabolites (Figures 5E and S7D). Specifically, KEGG enrichment analysis revealed significant activation of purine metabolism in DSS‐treated Setd2^vil‐ko^ mice (Figure 5F). The significantly altered metabolites within the purine pathway are detailed in Figure 5G. Notably, there was a profound elevation in uric acid, the terminal end product of purine catabolism (Figure 5G), suggesting an accelerated purine degradation rate in Setd2^vil‐ko^ mice.

### 3.6 Transplantation of healthy‐like gut microbiota decreases colitis susceptibility in SETD2‐deficient mice

**FIGURE 6:**
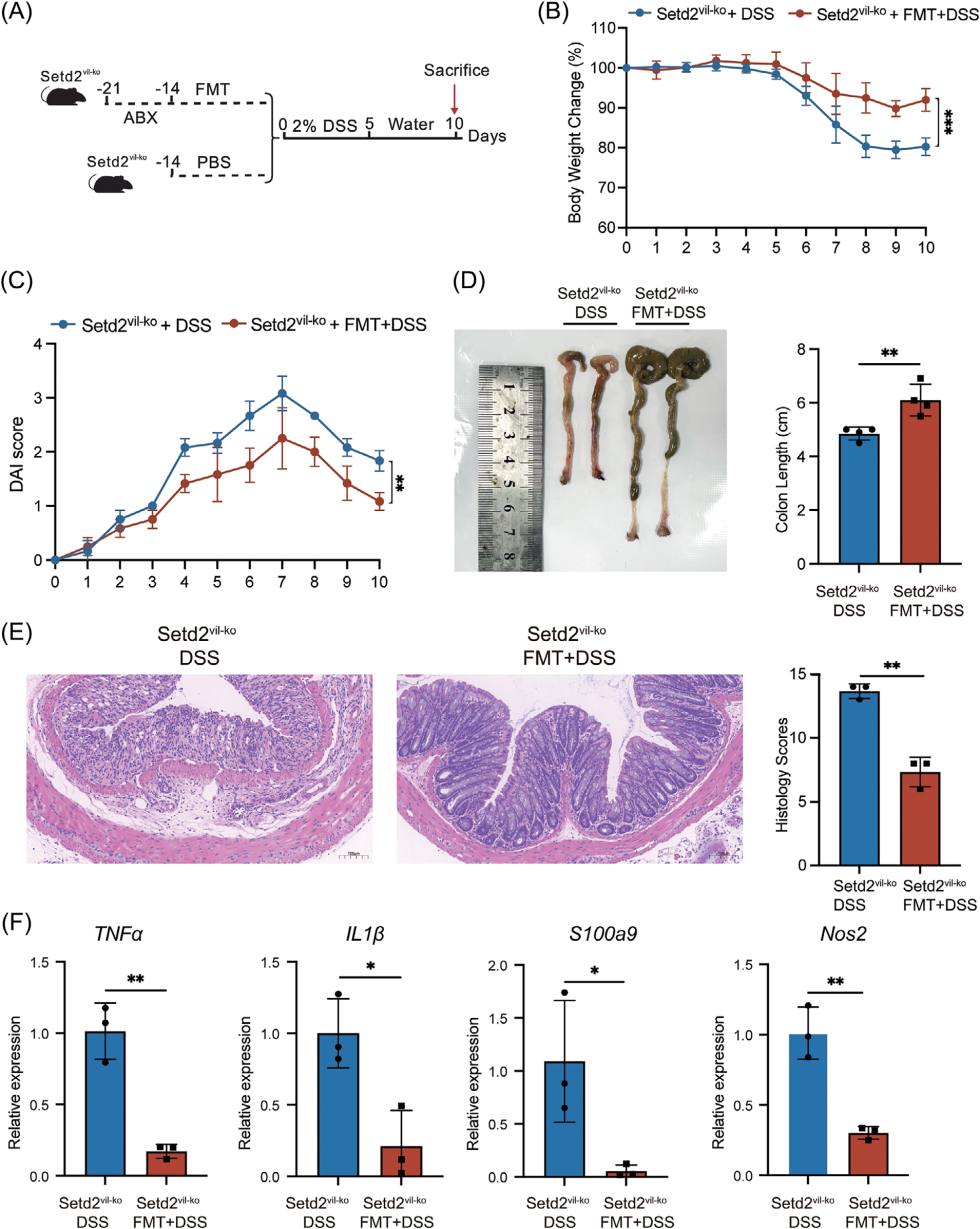
Transplantation of healthy‐like gut microbiota decreases colitis susceptibility in SET domain containing 2 (SETD2)‐deficient mice. (A) Schematic illustration of faecal microbiome transplantation (FMT). (B) Daily body weight changes in Setd2^vil‐ko^ mice with or without FMT. *n* = 4 mice per group. (C) Disease activity index (DAI) reflecting the inflammatory status. (D) Representative images and statistical analysis of colon shortening of Setd2^vil‐ko^ mice following FMT and DSS treatment. *n* = 4 mice per group. (E) Haematoxylin and eosin (H&E) staining of colon tissues collected on 10 days after the beginning of DSS treatment and corresponding quantification of histology scores (*n* = 3). (F) Relative mRNA expression levels of *TNFα*, *IL1β*, *S100a9* and *Nos2* determined by real‐time qPCR in in Setd2^vil‐ko^ mice with or without FMT. All the data are presented as mean ± standard deviation (SD). For body weight curve and DAI curve, *p*‐values were calculated at day 10. *p*‐Values were calculated using two‐tailed Student's *t*‐test. ^*^ *p* < .05; ^**^ *p* < .01; ^***^ *p* < .001; ^****^ *p* < .0001.

Given that SETD2 deficiency in IECs led to gut microbiota dysbiosis, we next investigated whether healthy‐like gut microbiota could alleviate experimental colitis caused by SETD2 deficiency. We performed FMT experiments to transfer gut microbiota from healthy wild‐type mice into ABX‐treated Setd2^vil‐ko^ recipients, with PBS‐treated Setd2^vil‐ko^ littermates serving as controls (Figure 6A). As expected, we found FMT‐treated mice exhibited marked clinical and histological improvement compared to PBS‐treated controls. Specifically, FMT‐treated mice showed significantly attenuated body weight loss during DSS challenge and lower DAI scores (Figure 6B,C). At the time of sacrifice, FMT‐treated mice displayed significantly longer colon lengths compared to controls (Figure 6D). Histopathological evaluation via H&E staining revealed that FMT treatment effectively protected the intestinal mucosal integrity, characterised by preserved crypt architecture and diminished infiltration of inflammatory cells, leading to a significant reduction in histopathological scores (Figure 6E). Consistently, qPCR analysis revealed that mRNA expressions of inflammatory markers (*TNFα*, *IL1β*, *S100a9* and *Nos2*) in FMT‐treated mice were profoundly suppressed (Figure 6F). Collectively, these data demonstrated that reconstruction of healthy gut microbiota could attenuate colitis susceptibility caused by SETD2 deficiency.

## 4 DISCUSSION

**FIGURE 7:**
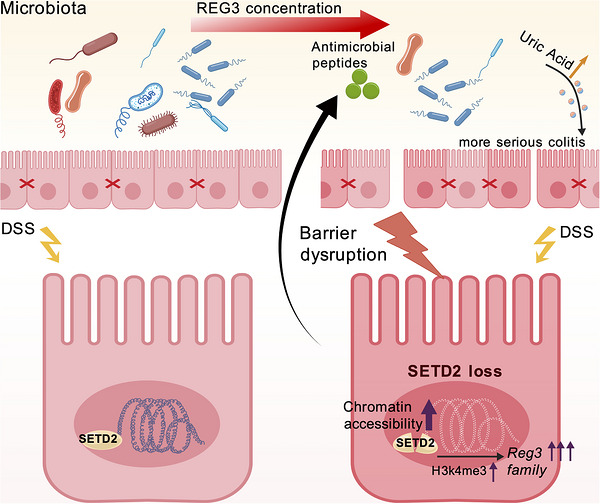
Schematic representation of the mechanism by which SET domain containing 2 (SETD2) maintains gut microbial homeostasis to attenuate colitis.

Epigenetic modifications are increasingly recognised as pivotal regulators of the crosstalk between the host organism and the composition or activity of its associated gut microbiota.^12^ As a key epigenetic regulator, SETD2 has been extensively characterised as a tumor suppressor in many malignancies,^8^, ^40^, ^41^ but its role in maintaining intestinal homeostasis remains largely unexplored. In this study, we discovered that SETD2 deficiency in IECs could induce the excessive expression of REG3 family genes. Based on the established antimicrobial functions of REG3 lectins, REG3 upregulation potentially disrupted beneficial commensal bacteria and caused a pathological accumulation of metabolites, thereby aggravating colitis (Figure 7).

REG3 lectins are regarded as essential guardians of intestinal homeostasis, enforcing spatial separation between the microbiota and intestinal epithelial surface, suppressing inflammation and preserving epithelial barrier integrity.^42^, ^43^, ^44^ Herein, we observed a striking upregulation of the AMPs, REG3 lectins, in Setd2^vil‐ko^ mice exhibiting more serious colitis. Integrated analysis of RNA‐seq and ATAC‐seq data revealed that SETD2 deficiency leads to significantly increased chromatin accessibility at the promoter and gene body regions of Reg3 loci. Interestingly, while SETD2 is a well‐known H3K36me3 methyltransferase, Cut&Tag‐seq failed to detect discernible H3K36me3 peaks at the Reg3 loci. Instead, higher levels of H3K4me3 were detected at the promoters of Reg3 genes in Setd2^vil‐ko^ mice. H3K4me3 is a pivotal epigenetic regulator well known for activating transcription^45^ and impaired H3K36me3 deposition might lead to abnormal over‐establishment of H3K4me3.^31^, ^32^, ^33^, ^34^ These findings indicate that SETD2 deficiency promotes Reg3 expression not through the conventional, direct reduction of H3K36me3, but rather via an indirect epigenetic remodelling mechanism. SETD2 deficiency may reshape chromatin landscape, allowing other histone methyltransferases or transcription factors to activate Reg3 expression. While REG3 lectins function as essential AMPs that maintain mucosal homeostasis under physiological conditions, they act as a double‐edged sword in the pathogenesis of IBD.^28^ Meanwhile, our data suggest that uncontrolled overproduction of REG3 under SETD2‐deficient conditions is detrimental rather than protective. Instead of selectively targeting pathogens, the AMPs surge may create a hostile environment that exerts indiscriminate antimicrobial pressure, potentially eliminating beneficial commensal bacteria that are crucial for maintaining microbial community stability.^35^, ^46^

Gut microbiota plays an important role in shaping the intestinal immune landscape and modulating host inflammatory responses. Upon depletion of gut microbiota via ABX, the exacerbated colitis phenotype previously observed in Setd2^vil‐ko^ mice was effectively reversed, indicating that gut microbiota is an indispensable driver of the pathological progression triggered by SETD2 deficiency. Our 16S rRNA sequencing data revealed that under homeostatic conditions, the overall microbial structures were similar between genotypes. However, DSS challenge induced a profound divergence, resulting in a severe restructuring of the gut microbiome characterised by a significant reduction in microbial diversity and a marked depletion of beneficial taxa, notably members of the *Muribaculaceae* and *Lachnospiraceae* families. *Muribaculaceae* and *Lachnospiraceae* are considered promising ‘next‐generation probiotics’ due to their established roles in regulating intestinal barrier function and immune responses.^47^, ^48^ Importantly, REG3 lectins are well‐known AMPs primarily responsible for regulating bacterial colonisation.^38^, ^39^ Consistently, Kini et al. found that the decrease of *Muribaculaceae* and *Lachnospiraceae* was accompanied by REG3 overexpression aligning with our observations.^37^ In addition, previous studies have reported that overproduction of REG3 lectins can be detrimental by reducing protective gut microbiota.^35^, ^36^ We thus speculated that under inflammatory stress, the excessive production of REG3 lectins induced by epithelial SETD2 deficiency may disrupt the gut microbial homeostasis. Furthermore, our metabolomic analysis provided important functional insights into these microbial shifts. Under homeostatic conditions, tryptophan metabolism was active but largely disappeared following DSS induction, potentially reflecting a decline in specific commensal populations, such as *Lachnospiraceae* and *Muribaculaceae*, that are actively involved in tryptophan catabolism.^49^, ^50^, ^51^, ^52^ Furthermore, we observed a marked accumulation of uric acid, the end product of purine metabolism in SETD2^vil‐ko^ mice, particularly during DSS challenge. Under homeostatic conditions, uric acid can be degraded by specific gut bacteria; however, the microbial dysbiosis induced by SETD2 deficiency likely impairs this catabolic capacity, leading to intestinal hyperuricaemia.^53^ The accumulation of uric acid can induce oxidative stress and increases intestinal barrier permeability, thereby exacerbating colitis.^53^, ^54^

Nevertheless, transplantation of healthy‐like gut microbiota into Setd2^vil‐ko^ mice significantly attenuated their heighted susceptibility to colitis and ameliorated intestinal inflammation. The rescue effect of FMT identifies microbial dysbiosis as the key driver of colitis exacerbation in the absence of SETD2 and suggests that restoring microbial homeostasis is a viable individualised treatment strategy for patients with IBD exhibiting reduced SETD2 expression in the intestine. While clinical validation remains essential, our data propose SETD2 expression status as a candidate predictive biomarker to guide personalised FMT application, thereby advancing precision medicine strategies for patients with IBD.

Despite these significant findings, there are several limitations in our study that warrant future investigation. First, our conclusions are based primarily on a mouse model of DSS‐induced colitis, validation in patients with IBD to confirm the regulation of SETD2 on REG3 is necessary. Second, the direct regulatory mechanisms connecting REG3, specific microbiota, and their downstream metabolites have not been fully elucidated. Future study requires in vivo experiments, such as using specific bacterial colonisation to observe direct changes in the metabolic profile. Additionally, in vitro assays are needed to investigate how these distinct bacteria and metabolites interact with the intestinal epithelium under REG3‐mediated stress. Finally, while we demonstrated a strong association between SETD2‐mediated REG3 elevation and gut dysbiosis, introducing a REG3‐deficient background into the DSS colitis model remains essential to definitively determine whether blocking REG3 signaling can rescue the colitis phenotype and restore microbial homeostasis. Future studies utilising Reg3^vil‐ko^ mice or Setd2/Reg3 double‐knockout models combined with targeted faecal microbiota transplantation are warranted to fully validate the therapeutic potential of this axis. Nevertheless, our work provides a foundational step in understanding the epigenetic control of the gut ecosystem.

## 5 CONCLUSIONS

In summary, our study uncovers a previously unrecognised role for SETD2 in the pathogenies of intestinal inflammatory diseases. We propose that SETD2's epigenetic regulation of REG3 lectins and its impact on gut microbes and metabolism are crucial for intestinal homeostasis. These findings underscore the pivotal role of host epigenetic regulation in shaping gut microbial composition and provide new insights into the development of epigenetic‐targeted therapies in IBD and other disorders associated with SETD2 deficiency.

## AUTHOR CONTRIBUTIONS

Jing Feng performed most of the experiments and wrote the manuscript. Ziyi Wang and Yue Xu performed data analysis and helped build animal models. Jinjin Peng, Che Xu, Qingxin Xie, Yuyi Li, Wangshuang Chen, Jiaqing Chen and Xingyu Wang assisted in some experiments. Xiangjun Meng and Li Li conceived the experimental concept and designed the experiments. Wei‐Qiang Gao interpreted the data and revised the manuscript. Xiangjun Meng, Li Li and Wei‐Qiang Gao supported the funding. All the authors contributed to the article and approved the submitted version.

## CONFLICT OF INTEREST STATEMENT

The authors declare they have no conflicts of interest.

## ETHICS STATEMENT

The experimental protocols involving animals were approved by the Animal Care and Use Committee of Shanghai Ninth People's Hospital (SH9H‐2023‐A849‐SB).

## Supporting information



Supporting Information

Supporting Information

## Data Availability

All data can be available from the authors upon reasonable request. The RNA‐seq raw data generated in this study has been deposited in the Gene Expression Omnibus (GEO) under accession number GSE318910. The ATAC‐seq, CUT&Tag‐seq and 16S rRNA‐seq raw data have been deposited in the Genome Sequence Archive (GSA) in the National Genomics Data Center, Beijing Institute of Genomics (China National Center for Bioinformation), Chinese Academy of Sciences, under accession number CRA038677, CRA045976, CRA038804 (DSS‐treated) and CRA046015 (water‐treated). Bulk RNA‐seq datasets (GSE207022 and GSE9452) were obtained from GEO. Our former scRNA‐seq dataset has been deposited in GSA (HRA007064).^25^
