# Supplementary material for: Intestinal epithelial SETD2 maintains gut microbial homeostasis to attenuate colitis
Source: Clin Transl Med. 2026 Aug 4;16(8):e70754. doi: 10.1002/ctm2.70754 (PMC13434441; doi:10.1002/ctm2.70754)
Supplement: Supplementary file 1 [file CTM2-16-e70754-s002.pdf]

Figure S1. Confirmation of IEC-specific knockout of SETD2.

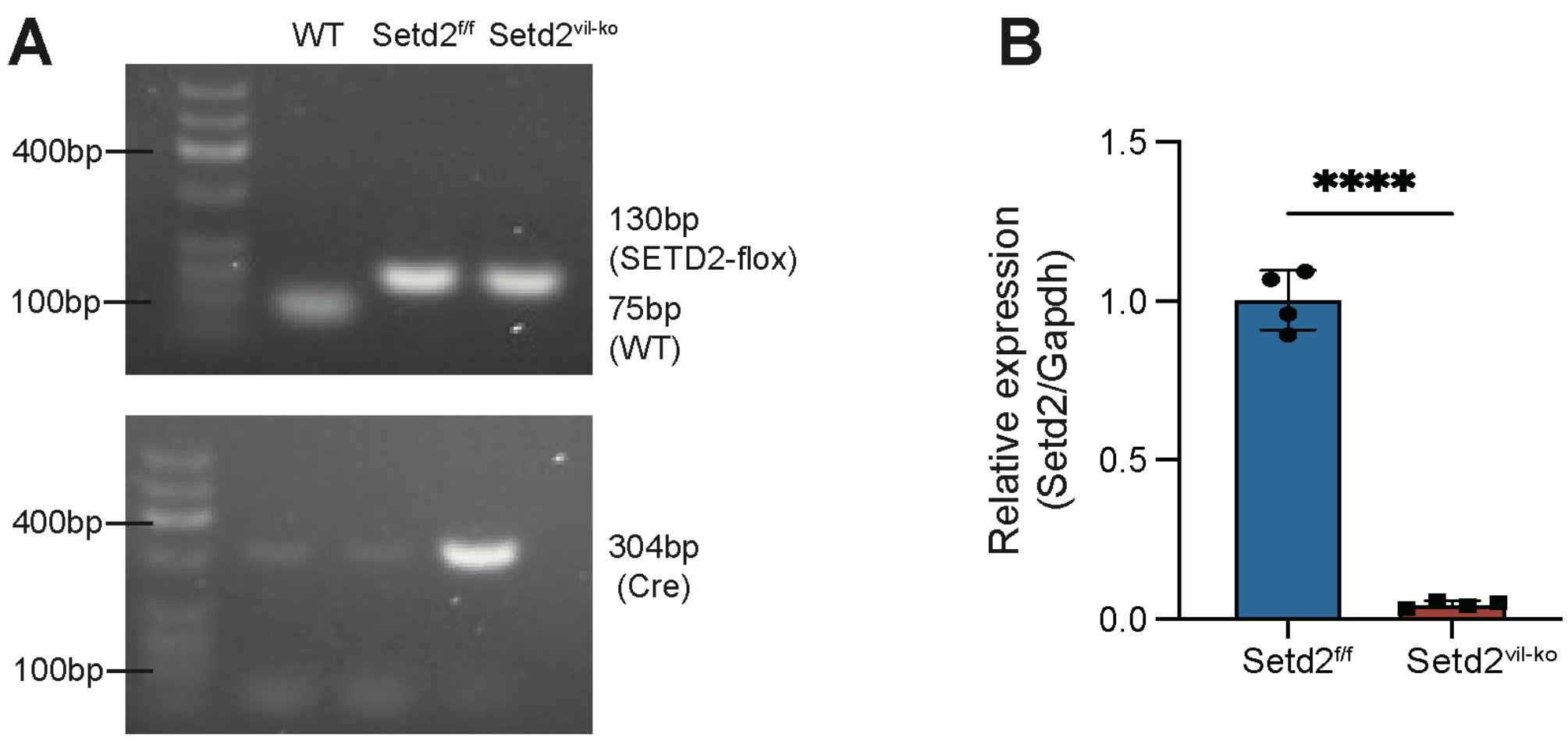

(A) PCR validation of SETD2 conditional knockout mice. (B) *Setd2* mRNA expression in IECs isolated from Setd2$^{f/f}$ and Setd2^{vil-ko} mice.

Figure S2. Representative images of colon shortening and statistical analysis in water-treated mice.

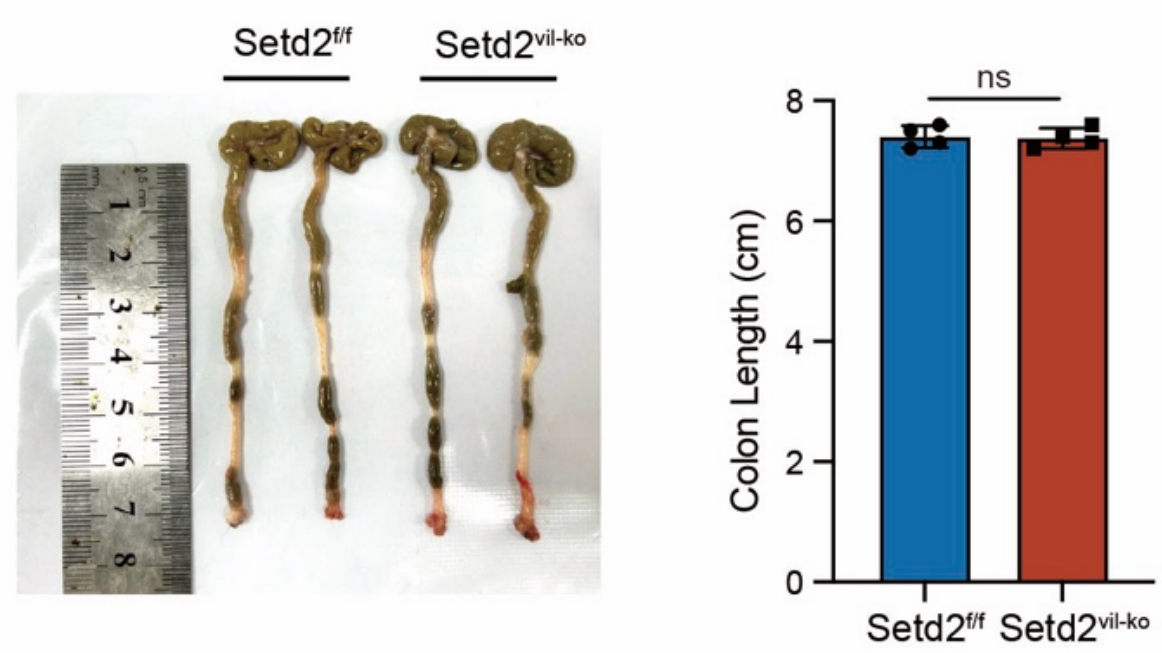

Figure S3. Immunofluorescence staining for EPCAM and REG3 proteins.

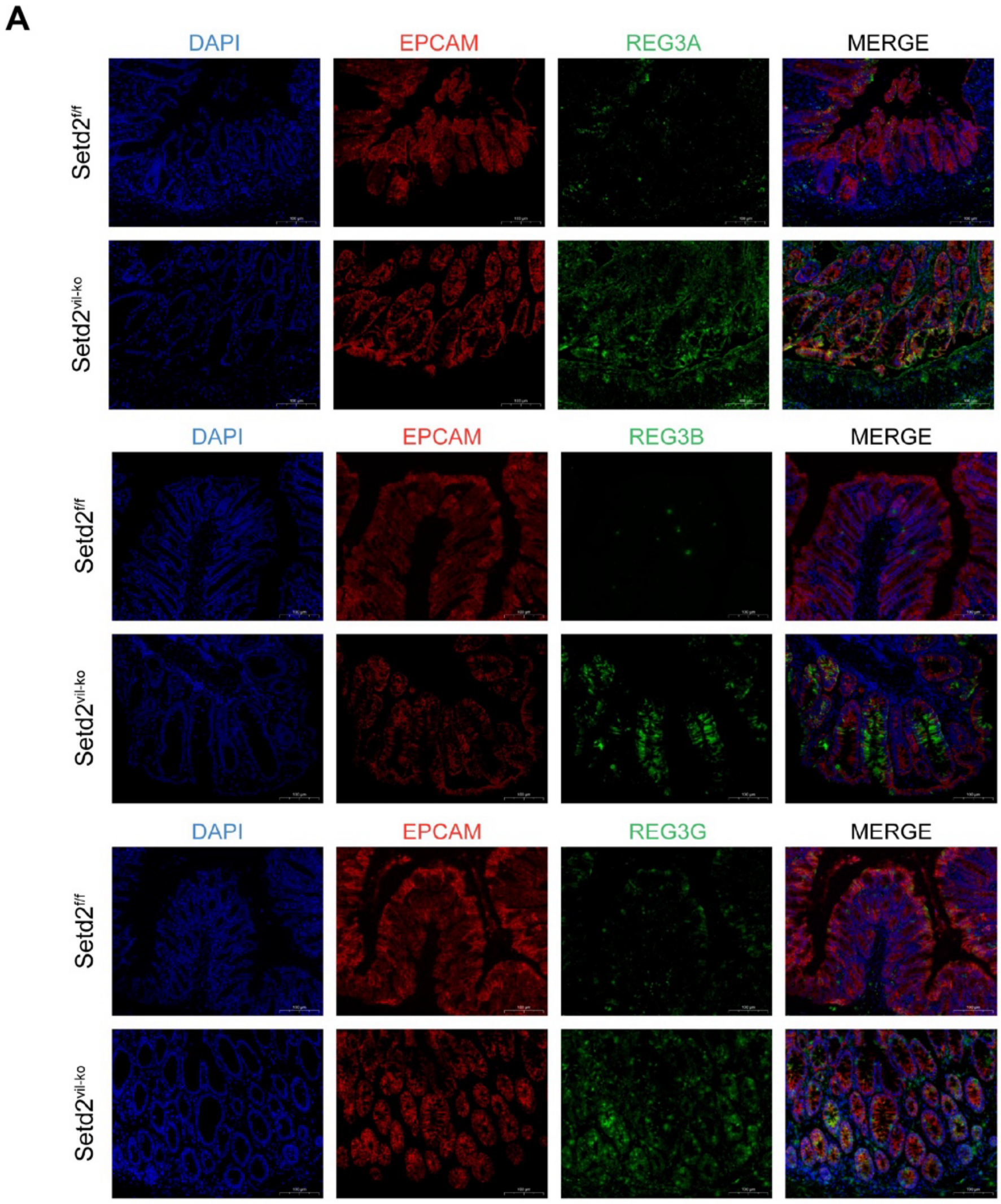

Figure S4. SETD2 knockout efficiency in HT-29 cells was confirmed by qPCR analysis.

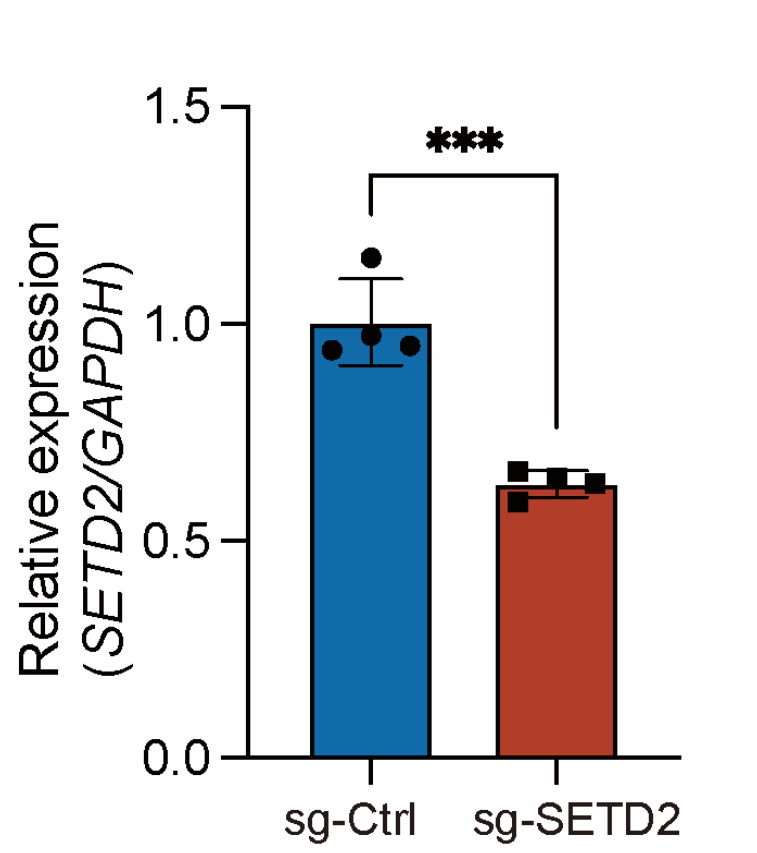

Figure S5. Enrichment profiles of H3K36me3 (A) and H3K4me3 (B) around peak centers.

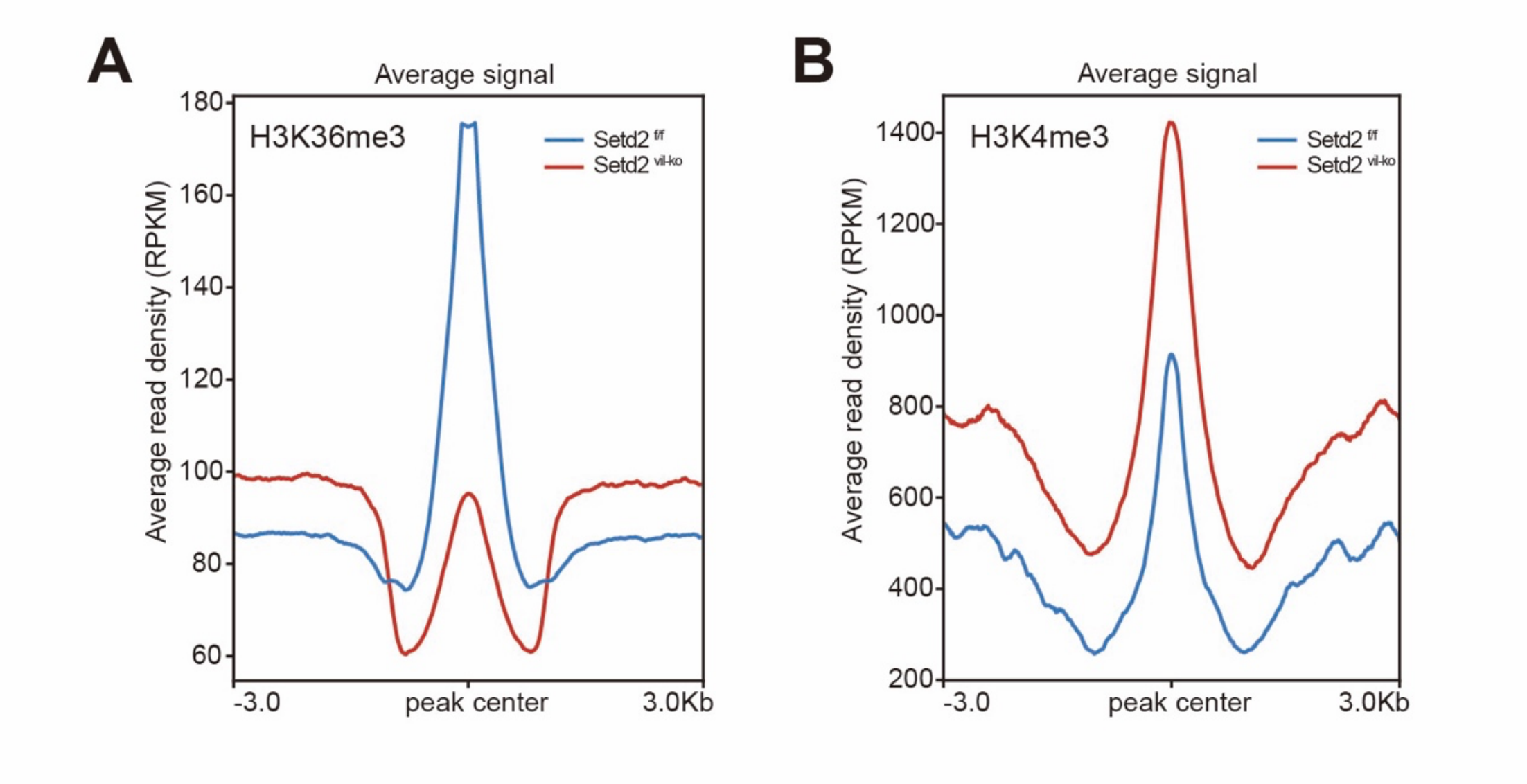

Figure S6. Gut microbiota dysbiosis is indispensable for SETD2 deficiency-induced colitis exacerbation.

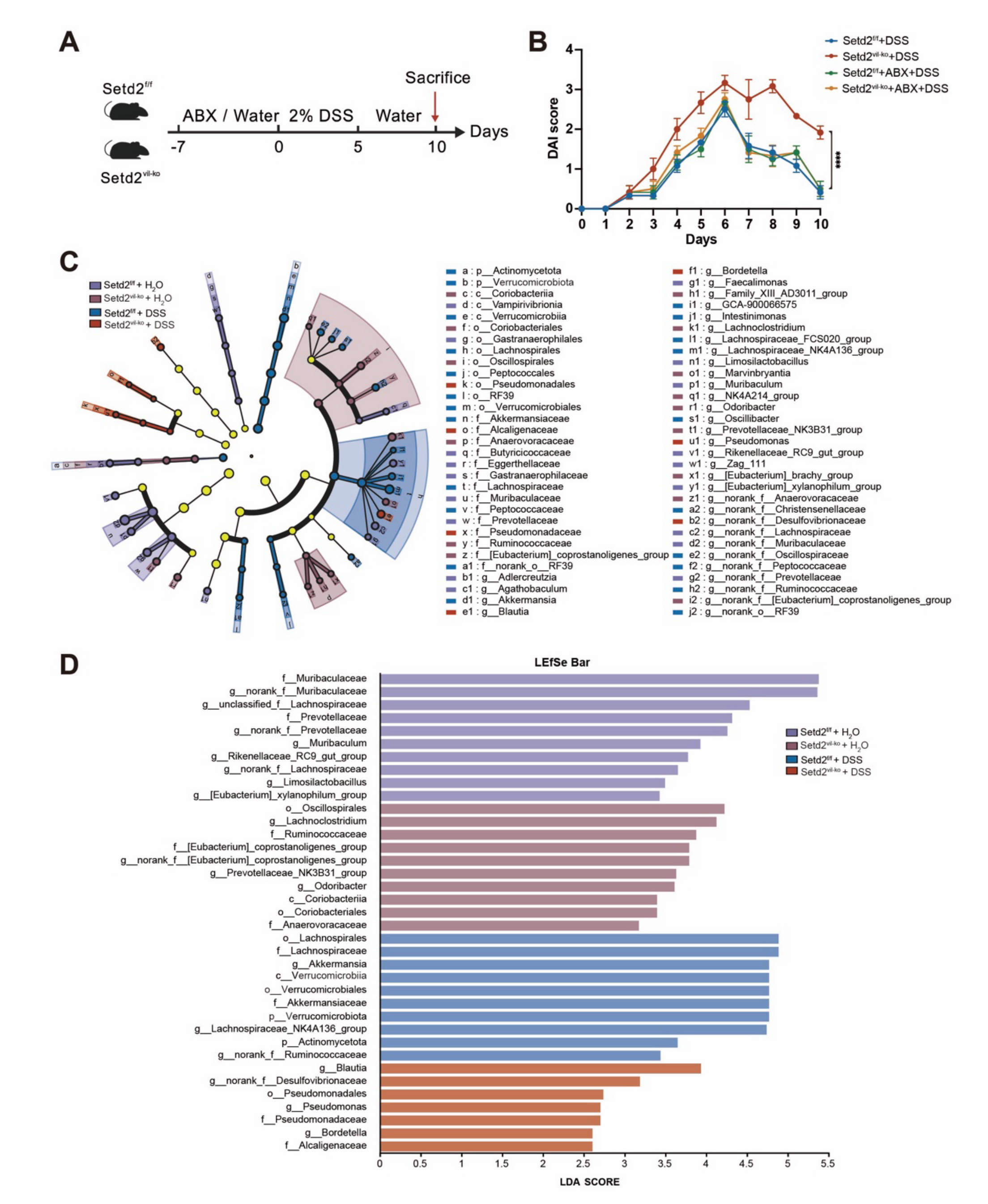

(A) Schematic illustration of the DSS-induced colitis in ABX-treated or untreated Setd2$^{f/f}$ and Setd2^{vil-ko} mice. (B) Disease activity index (DAI) reflecting the inflammatory status. (C) LEfSe cladogram depicting dominant microbial taxa differentiating the four groups from phylum to genus level. LDA score > 2 were displayed. (D) LEfSe analysis revealed the dominant microbial taxa at the family level. Taxa with an LDA score > 2 were considered to significantly contribute to the observed intergroup differences in microbial abundance.

Figure S7. SETD2 deficiency in IECs alters fecal metabolites during colitis.

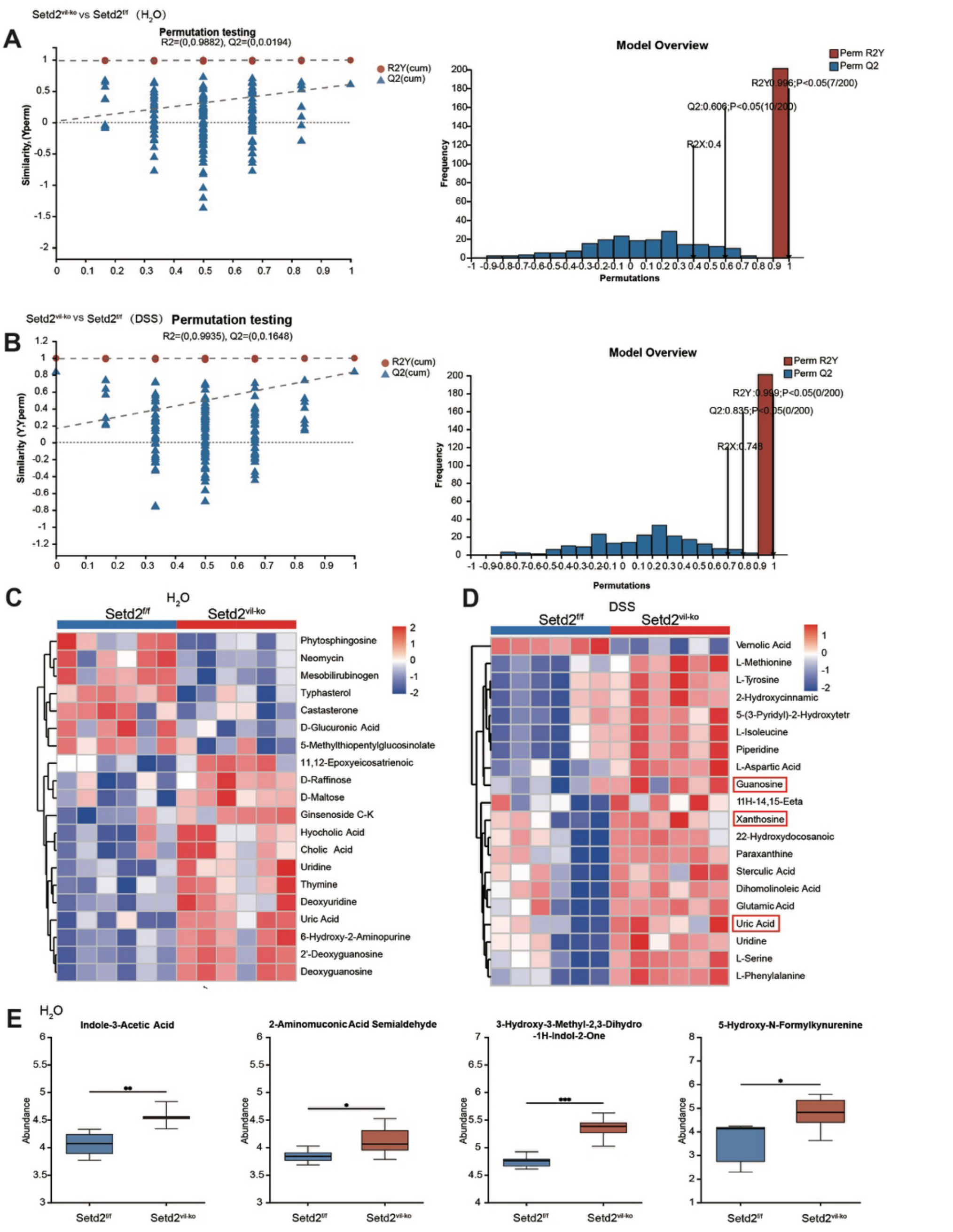

(A-B) Orthogonal partial least squares-discriminant analysis (OPLS-DA) model validation for water-treated mice (A) and DSS-treated mice (B). Each panel displays the permutation test scatter plot (left) and the corresponding permutation test histogram (right) to assess model predictability and prevent overfitting. (C-D) Heatmap of the top 20 discriminating metabolites by abundance for water-treated mice (C) and DSS-treated mice (D). (E) Comparison of the relative abundance of tryptophan metabolites in water-treated mice.
