# Supplementary material for: Intestinal epithelial SETD2 maintains gut microbial homeostasis to attenuate colitis
Source: Clin Transl Med. 2026 Aug 4;16(8):e70754. doi: 10.1002/ctm2.70754 (PMC13434441; doi:10.1002/ctm2.70754)
Supplement: Supplementary file 2 [file CTM2-16-e70754-s001.docx]

| **Table S1. Primer sequences used for mouse genotyping analysis.** | |
| --- | --- |
| Primer | Sequence |
| SETD2-FLOX-F | GTAAAGTAGTATTATGCCAAGGCCC |
| SETD2-FLOX-R | TATTTAAACTCTCTCTGGGGGTGG |
| villin-F | GTGTTTGGTTTGGTTTCCTCTGCATAAGA |
| cre-R | GCAGGCAAATTTTGGTGTACGGTCA |

| **Table S2. Primers and Oligonucleotides Sequences for qPCR** | | | |
| --- | --- | --- | --- |
| Gene | Species | Forward primer | Reverse primer |
| *TNFα* | Mouse | CCACGCTCTTCTGTCTACTG | GCTACGGGCTTGTCACTC |
| *IL1β* | Mouse | GAAATGCCACCTTTTGACAGTG | TGGATGCTCTCATCAGGACAG |
| *S100a9* | Mouse | ATACTCTAGGAAGGAAGGACACC | TCCATGATGTCATTTATGAGGGC |
| *Nos2* | Mouse | ACATCGACCCGTCCACAGTAT | CAGAGGGGTAGGCTTGTCTC |
| *Defa24* | Mouse | CTAAAACTGAGGAGCAGCCAGG | CTTCCTTTGCAGCCTCTTGCTC |
| *Adgrb1* | Mouse | CTCCACCATTGATGTCCTGAGG | TCTTCTGCCAGCAGGTTGCTGA |
| *Slpi* | Mouse | GGCCTTTTACCTTTCACGGTG | TACGGCATTGTGGCTTCTCAA |
| *IL17a* | Mouse | TTTAACTCCCTTGGCGCAAAA | CTTTCCCTCCGCATTGACAC |
| *Cxcl5* | Mouse | TCCAGCTCGCCATTCATGC | TTGCGGCTATGACTGAGGAAG |
| *Reg3a* | Mouse | GGGTTACAAGGCTTATCGCTCC | GGACACAAAGGAAGCCTCACCT |
| *Reg3b* | Mouse | ACTCCCTGAAGAATATACCCTCC | CGCTATTGAGCACAGATACGAG |
| *Reg3g* | Mouse | ATGCTTCCCCGTATAACCATCA | GGCCATATCTGCATCATACCAG |
| *Setd2* | Mouse | GCCTTCGTGTGCTATGGATGAC | TCACACTGCATTCGCTTAATATCC |
| *Gapdh* | Mouse | AGGTCGGTGTGAACGGATTTG | AGGTCGGTGTGAACGGATTTG |
| *GAPDH* | Human | AGATCCCTCCAAAATCAAGTGG | GGCAGAGATGATGACCCTTTT |
| *REG3A* | Human | TAATGTGAGGTTACCCTATG | GAGGAAGAAACAGAAGAAAG |
| *REG3G* | Human | GGTGAGGAGCATTAGTAACAGC | CCAGGGTTTAAGATGGTGGAGG |
| *SETD2* | Human | GGCAGAGATGATGACCCTTTT | GGCAGAGATGATGACCCTTTT |
